# Does Closeness Make the Heart Grow Weaker?: Heart Attacks and Proximity to Local Traffic

**Published:** 2007-01

**Authors:** Bob Weinhold

Growing evidence links heart attacks with short-term exposures to vehicle exhaust from nearby streets. Now some of the first evidence that long-term exposures also are a culprit has been published by a team of Massachusetts researchers **[*EHP* 115:53–57; Tonne et al.]**. Cardiovascular disease, of which heart attacks are one major type, is the leading killer in the United States and much of the world.

The team evaluated more than 5,000 cases of acute myocardial infarction that occurred in residents of the mid-sized city of Worcester, Massachusetts, from 1995 to 2003, to determine if there was any connection between the heart attacks and exposure to traffic. They used two measures of exposure: cumulative local traffic within 100 m of the home, and the distance of the individual’s house to major roadways. They also factored in variables such as age, sex, income, education, amount of open space in the town, and nearby point sources of fine particulates.

They found that local traffic within 100 m of an individual’s house was associated with a 4% increase in heart attack risk for each interquartile increase in cumulative traffic volume. The linkage wasn’t as strong at 200 m and 300 m, which fits with other findings that local traffic-related pollutants tend to diminish around 100–150 m from the roadside. For major roadways (such as highways), heart attack risk increased by 5% for each kilometer closer to the road.

For unknown reasons, there was a link to individuals’ age, with those under 65 being most vulnerable. Those aged 65 to 74 were less vulnerable, and there was no link for those aged 75 and older. There also were significant links between increasing heart attack risk and both decreasing open space and increasing poverty.

The high number of cases studied is one of the many strengths of the study. However, the authors acknowledge that a number of factors need to be better studied to more fully understand what is going on. For instance, they were unable to study individual income and education. Instead, they relied on census block group data, which reflects some homogeneity of socioeconomic conditions, but doesn’t capture individual variations. Other weaknesses included a reliance on estimates instead of actual counts for local traffic volumes, and lack of personal pollution exposure data. In addition, these cases represent circumstances in just one city and one period of time. Nonetheless, the study offers some of the first evidence that long-term exposure to vehicle emissions may be an important contributor to heart attacks.

## Figures and Tables

**Figure:**
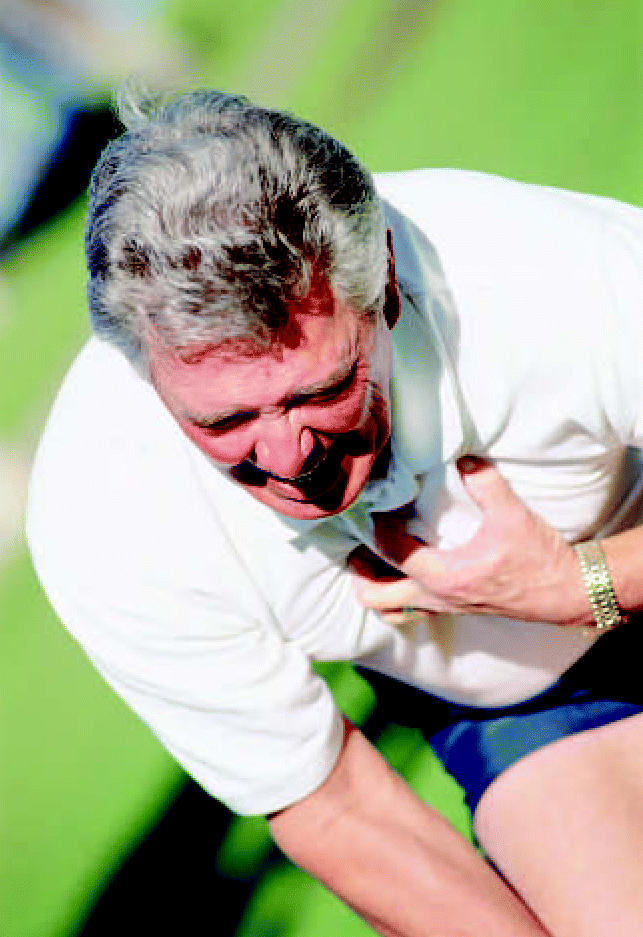
Hazard near home. New data link small increases in heart attack risk to living within 100 m of local traffic and major roads.

